# Exercise and Bone Health in Cancer: Enemy or Ally?

**DOI:** 10.3390/cancers14246078

**Published:** 2022-12-10

**Authors:** Alice Avancini, Giulia Benato, Anita Borsati, Luca Oliviero, Lorenzo Belluomini, Marco Sposito, Daniela Tregnago, Ilaria Trestini, Jessica Insolda, Francesca Zacchi, Elena Fiorio, Federico Schena, Michele Milella, Sara Pilotto

**Affiliations:** 1 Section of Oncology, Department of Medicine, University of Verona School of Medicine and Verona University Hospital Trust, 37134 Verona, Italy; 2 Department of Neurosciences, Biomedicine and Movement Sciences, University of Verona, 37131 Verona, Italy; 3 Dietetics Service, Medical Direction, University Hospital of Verona, 37134 Verona, Italy

**Keywords:** exercise, bone metastases, bone loss, bone remodeling, cancer disease

## Abstract

**Simple Summary:**

Patients with cancer may face bone metastases and osteoporosis due to cancer or treatments, leading to a high risk of developing skeletal-related events. Skeletal-related events may negatively affect patients’ quality and length of life. Although physical exercise has been recognized as a potential adjunctive strategy in the cancer setting, it is often not recommended to patients with bone health impairments due to safety concerns. In the present review, we explore the effects of exercise on safety profile, bone health, and the impact on functional outcomes in patients with cancer affected by bone metastasis, osteoporosis/osteopenia, or at high risk of losing bone. Moreover, the underlying mechanisms of the beneficial effect of exercise on bone are explored, and considerations about exercise prescription are discussed.

**Abstract:**

Bone health is often threatened in cancer patients. Bone metastasis and osteoporosis frequently occur in patients with cancer and may lead to different skeletal-related events, which may negatively affect patients’ quality of life and are associated with high mortality risk. Physical exercise has been recognized as a potential adjunctive strategy in the cancer setting to improve physical function as well as treatment-related side effects. Nevertheless, exercise is often not recommended to patients with bone health impairments due to safety concerns. In the current review, we aimed, through a comprehensive review of the evidence, to explore the impact of exercise in terms of safety profile, bone outcomes, and the effects on other outcomes in patients with cancer affected by bone metastasis or at high risk of losing bone. Additionally, we explored the potential mechanisms by which exercise may act on bone, particularly the impact of mechanical load on bone remodeling. Finally, considerations about exercise prescription and programming in these populations are also discussed.

## 1. Introduction

Bone health is often threatened in patients with cancer. Cancer and its treatments may damage the skeleton, exposing patients to an increased risk of skeletal-related events (SREs).

Bone metastasis is, firstly, a frequent complication in solid tumors, occurring most frequently in advanced prostate (85%), breast (70%), lung (40%), and kidney (40%) malignancies [1]. The spine, pelvis, skull, ribs, proximal humeri, and femora are the areas most affected by the metastatic spread, probably reflecting the distribution of the red bone marrow [1]. Bone metastases may be classified as osteoblastic, osteolytic, or mixed. Osteoblastic bone metastases, developing through the stimulation of osteoblast proliferation and differentiation, are associated with the deposition of new pathological bone and are typically observed in prostate cancer [2]. On the other hand, osteolytic metastases, often occurring in breast, lung, and renal cancers, are characterized by osteocyte activation, thus resulting in the destruction of normal bone [2]. In some cases, bone metastases may be mixed, i.e., with both components of osteoblastic and osteolytic lesions [2]. Bone metastases expose patients to a high risk of SREs, such as pathological fracture, the need for radiotherapy and/or surgery to bone, spinal cord compression, and hypercalcemia. Skeletal morbidities are reported in lung (53.4%), prostate (45.9%), and breast (43.6%) cancers with bone involvement [3] and occur more frequently in patients with osteolytic lesions [4]. Different treatment options are available, including radiotherapy, surgery, bone-targeted agents (i.e., bisphosphonates and denosumab), as well as systemic treatment for the underlying oncological disease. Nevertheless, these approaches have a major role only in preventing disease progression and palliating symptoms [5].

On the other hand, patients affected by early stage cancer may be subjected to an increased loss of bone mineral density, leading to developing osteoporosis, i.e., a systemic disorder characterized by low bone mass leading to bone fragility. A prospective study revealed a prevalence of 16% for osteoporosis and 44% for osteopenia (a loss of bone density not so severe as osteoporosis) among 1041 patients affected by different malignancies [6]. The peak bone mass, usually reached around 30 years, is the major determinant of an individual bone density [7], which begins to decline due to age and changes in sex-steroid hormones [7]. Beyond genetic predisposition, several lifestyle factors may accelerate bone loss, such as tobacco smoking, high alcohol consumption, impaired mobility, low body weight, nutritional deficiencies (e.g., calcium intake), and low physical activity [8]. Additionally, some drugs, e.g., corticosteroids, and different anticancer treatments, including gonadotropin-releasing hormone (GnRH) agonists, chemotherapy-induced ovarian failure (CIOF), aromatase inhibitors (AIs), and androgen-deprivation therapy (ADT), may decrease bone mineral density [8]. For instance, patients with non-metastatic prostate cancer treated with ADT experience a reduction in bone mineral density ranging from 2.29% to 5.5% during the first year of treatment, which continues throughout the second year at a slower rate [9]. Similarly, patients in postmenopausal status undergoing AIs may report a decrease in bone mass of around 2–3% per year, whereas premenopausal women lose bone by approximately 7 and 7.7% due to CIOF and GnRH agonists, respectively [10]. Despite bone-targeted agents, as well as positive changes in lifestyle factors associated with the risk of osteoporosis may help slow down bone loss [8], patients undergoing the aforementioned anticancer treatments have an elevated risk of fracture [10,11].

SREs related to bone metastases and fractures related to osteoporosis may impair patients’ quality of life and are associated with an increased risk of mortality [12,13] and a high economic burden [14]. In this scenario, strategies addressed to preserve physical function, improve patients’ quality of life, decrease the risk of falls, as well as improve bone health and, thus, diminish the risk of SREs and fractures, are crucial.

Physical activity and exercise have been proven to be beneficial in the oncological setting. Observational evidence suggested a positive association between physical activity and survival, especially in breast, colorectal, and prostate cancers [15]. Additionally, randomized controlled trials have demonstrated the beneficial effects of exercise in improving physical function through an increase in cardiorespiratory fitness [16], muscle strength [17], and optimization of body composition [17]. Exercise intervention may help to enhance the quality of life and ameliorate some side effects of cancer and its treatments, such as fatigue, peripheral neuropathy, and lymphoedema; moderate evidence is available for bone health [18]. Regarding bone health, exercise may be an important tool for improving bone remodeling, matrix mineralization, and marrow health, thus leading to the preservation of bone mineral density [19,20]. Nevertheless, despite the well-known benefits of exercise, some concerns about safety issues in patients suffering from bone fragility may arise. In this sense, a survey found that about 40% of oncology providers working in lung cancer settings report having no opinion or agree that exercise should be avoided in patients with bone metastases [21]. Similarly, another study involving oncologists and palliative care physicians shows that 65% are worried about a potential increase in fracture risk due to physical activity in patients with metastatic bone disease, osteoporosis, or undergoing ADT [22]. In contrast, the large majority of patients with bone metastases consider it important to be physically active, they feel able to exercise, and are interested in participating in exercise programs [23]. Although prior reviews have investigated the effect of exercise in patients with bone metastases or those at risk of bone loss in separate studies [24,25], a comprehensive review including both aspects is currently missing. With these premises, the present review aims to elucidate the impact of exercise in patients with bone metastases and those with or at risk of developing osteoporosis regarding safety, efficacy on bone health, psychological well-being, and health-related fitness components, such as cardiorespiratory fitness, strength, and body composition. Additionally, the potential mechanisms by which exercise could remodel bone mass and considerations about exercise prescription in these populations are also summarized.

## 2. Materials and Methods

To explore the role of exercise in patients with cancer affected by bone metastases or osteoporosis at risk of bone loss, a comprehensive search on PubMed (MEDLINE), Cochrane Central Register of Controlled Trials (CENTRAL), Scopus, and SPORTDiscus was performed. The following keywords drove the research: “metastatic cancer”, “bone metastases”, “osteoporosis”, “osteopenia”, “bone health”, “exercise”, “physical activity”, “physical exercise”. Trials were included if they had a randomized controlled design; included patients with cancer affected by bone metastasis or those with osteoporosis/osteopenia or at high risk of developing it; and investigated the effect of exercise as a form of planned, structured, and repetitive body movement to improve physical fitness components. Published abstracts, non-full text, non-English articles, and interventions involving general physical activity recommendations were excluded. Two independent reviewers screened the literature (G.B. and L.O.). Disagreements were discussed and resolved by a third reviewer (A.A.).

## 3. Exercise and Bone Metastasis

A series of investigations have explored the impact of exercise on different outcomes, including safety profile, effect on bone mass, and other parameters, in patients with bone metastases (Table 1).

### 3.1. Safety of Exercise

The National Cancer Institute defines an adverse event (AE) as an “unfavorable and unintended sign (including an abnormal laboratory finding), symptom, or disease temporally associated with the use of a medical treatment or procedure that may or may not be considered related to the medical treatment or procedure” [49]. Based on this definition, AEs in studies testing exercise can be categorized as non-exercise-related AEs, i.e., unrelated to exercise intervention, and exercise-related AEs, i.e., occurred during the exercise sessions. One investigation did not measure safety [42]. Whereas eight trials did not record any AEs during the exercise period [26,27,28,29,30,31,36,39,43,45,48,50], two investigations reported exercise-related AEs [33,34,35,44], two described non-exercise-related AEs [38,41], and four recorded both exercise and non-exercise-related AEs [37,40,46,47]. Among them, only two studies reported serious AEs related to exercise training [33,34,40]. Concerning non-exercise-related AEs, the number of reported side effects appears similar among patients who engage in an exercise intervention compared to the controls [37,38,40,41]. On the other hand, most AEs occurring during the exercise sessions were classified as non-serious, such as fatigue, back pain, dizziness, and muscle strain, whereas only two studies have associated exercise with serious SREs [33,34,35,40,41,44]. For instance, Uth and colleagues, in their trial which tested soccer in a sample of 57 patients with advanced prostate cancer (19.3% of them with bone metastasis), reported two fibula fractures and one partial rupture of the Achilles tendon during the training [33,34,35]. Nevertheless, whether these AEs occurred in patients with or without bone metastases is unclear. A second investigation on 214 men with prostate cancer (19% with bone metastases) described two ruptures of the Achilles tendon associated with exercise [40]. In this case, however, a sub-analysis revealed that those side effects occurred in patients without skeletal metastasis, thus excluding the possible association with bone disease [46]. Of note, focusing on the type of exercise, no serious SREs were observed in trials investigating resistance training as part of the exercise sessions.

Overall, the available data support the safety profile of exercise in patients with cancer affected by bone metastases, even in those interventions which included resistance training, an activity traditionally considered at high risk for fracture. However, some considerations are mandatory. Firstly, patients included in the current investigations might be highly selected and, thus, not fully representative of the entire cancer population with bone metastases. In this sense, most studies are addressed to patients with prostate and breast cancers, whereas limited or no information for other cancer types, such as lung or kidney, is available. Additionally, inclusion criteria for selecting patients with bone metastases rarely report detailed information and often exclude the frailest patients, such as those with bone pain or unstable metastases [26,27,33,39,44,45]. Secondly, the adopted criteria for monitoring and reporting AEs are sometimes not specified or heterogeneous across the studies. The introduction of a standardized classification may help to improve the accuracy of AE monitoring, which is fundamental to adequately assess safety, while preserving patient’s safety within a clinical trial. Future investigations should address these gaps in order to definitely consolidate the safety profile of exercise in patients with cancer affected by metastatic bone disease.

### 3.2. Effect of Exercise on Bone Health

In healthy subjects, physical exercise is a recognized lifestyle component able to maximize bone development, and improve and preserve bone health across the lifespan [51,52]. On the other hand, whether or not exercise may harbor the same benefits in patients with bone metastasis is still a significant subject of debate.

The available studies in this setting show mixed results [29,34,35,41,46,47]. *Bjerre and colleagues* found no significant differences in total hip and spine bone mineral density after 6 months of soccer training in 41 patients affected by prostate cancers with skeletal metastases [46]. Another similar investigation has explored bone adaptation to soccer training in 57 patients with advanced or metastatic prostate cancer (19.3% of them with bone metastases). Whereas post-intervention evaluations did not detect improvements in total body and leg bone mineral density, the bone mineral content of the leg (mean difference 13.8 g, 95% CI: 7.0 to 20.5 g) and total (mean difference 26.4 g, 95% CI: 5.8 to 46.9 g) statistically increased in the experimental group compared to the controls, thus suggesting a possible response in bone tissue after an exercise intervention [34]. Beyond the systemic impact on bone quality, exercise may directly affect the bone lesion, potentially contributing to its remineralization. In this sense, a randomized controlled trial testing isometric resistance exercise did not show significant differences in the density of the metastatic bone or pathological fracture rate in 60 patients affected by unstable spinal metastases and undergoing palliative radiotherapy. However, this study is characterized by a short survival in both groups (mean 4.4 months), leading to a high dropout rate (73% in the experimental group and 63% in the controls), which makes it difficult to know if the lack of results in bone outcomes are attributable to the small sample size or to exercise ineffectiveness [43]. Another similar investigation has compared the effect of exercise on metastatic bone density during radiotherapy in patients with stable spinal metastases [29]. Sixty patients were randomized to receive passive muscle therapy (controls), or isometric resistance training performed five days per week over two weeks and then three times per week until six months. Compared to controls that remained stable, the experimental group reported an improvement in bone density in all spine metastases, which significantly increased by 28.3% and 80.3% after three and six months, respectively. A sub-analysis by metastasis types revealed that, while no differences emerged from osteoblastic lesions, osteolytic metastases seemed to benefit more from exercise, increasing their density by about 88.8% and 179.3% after three and six months [29]. Moreover, biochemical evaluations found significant enhancements in bone turnover markers, especially pyridinoline and C-terminal cross-linking telopeptide of type I collagen, in the experimental group [32], further strengthening the hypothesis that exercise might be an adjunctive strategy able to produce a synergistic effect on radiotherapy to improve the recalcification of metastases.

### 3.3. The Overall Effect of Exercise 

Across the studies including patients with bone metastases, other outcomes, such as physical function, treatment-related side effects, and quality of life, have been investigated. Most of the investigations reported improvements in cardiorespiratory fitness and muscle strength [26,36,37,38,39,44,45,47], whereas the results on body composition appear more debated [33,34,36,38,39,44,46,47]. For instance, *Cormie* et al., in a randomized controlled trial in patients with bone metastatic prostate cancer, observed that 12 weeks of resistance training at moderate intensity twice a week was able to improve muscle strength, aerobic capacity, and lean body mass, whereas no effect in fat mass was detected [26]. On the contrary, a similar study combining aerobic and resistance training for three months in patients with metastatic bone disease confirmed a positive increase in strength and cardiorespiratory function but did not find any significant changes in lean and fat mass [36].

Regarding patient-reported outcomes, more than half of the studies did not report improvement in quality of life, distress, and fatigue levels, nor did they report negative effects [26,36,37,38,40,41,43,44], while other investigations suggest a possible positive impact on these outcomes [27,39,42,45,46]. Intriguingly, pain level has also been monitored. *Rief and colleagues,* in their trial assessing resistance training in patients with spinal bone metastasis, observed that exercise was able to relieve pain levels and reduce the oral morphine dose, as well as the concomitant non-opioid analgesics over six months [53]. Another three-arm randomized controlled trial including 516 patients with mixed cancer types (51.3% with bone metastases) has compared controls (arm 1) versus telerehabilitation (arm 2) (composed of walking-based program and resistance activities) and telerehabilitation plus pharmacological pain management (arm 3). After six months, compared to controls, both interventions exhibited equal effectiveness in improving pain interference (arm 2, −0.4: 95% CI: −0.78 to −0.09; arm 3, −0.4: 95% CI: −0.79 to −0.10) and intensity (arm 2, −0.4: 95% CI: −0.78 to −0.07; arm 3, −0.5: 95% CI: −0.84 to −0.11). Additionally, the total hospital days (335 days for arm 1 vs. 213 days for arm 2 vs. 284 days for arm 3) and the length of stay (7.4 days for arm 1 vs. 3.5 days for arm 2 vs. 5.0 days for arm 3) were lower in experimental groups than the control arm [42]. Since pain is one of the most impactful consequences of bone metastases, seriously affecting patients’ independence and quality of life, exercise may be considered a non-pharmacological adjunctive therapy with a potential analgesic effect in this setting.

## 4. Exercise and Bone Loss

Different studies have investigated the role of exercise in both patients with non-metastatic disease at high risk of losing bone and in those with a recognized bone fragility condition, i.e., affected by osteopenia or osteoporosis (Table 2).

### 4.1. Safety of Exercise

Although most investigations have not assessed the presence or absence of AEs [54,55,57,58,59,62,63,66,68,70,72], the reported findings support the safety profile of exercise [56,61,62,64,65,67,69,71,73,74,75]. In trials including patients with cancer at high risk of accelerated bone loss, e.g., those undergoing chemotherapy, endocrine therapy, or in postmenopausal status, the majority did not find any serious AEs [56,62,64,65,69,71,74], while three registered mild side effects [61,74,75]. For instance, *Nikander and colleagues,* in their randomized controlled trial, which consisted of a 12-month exercise intervention involving patients with breast cancer undergoing endocrine therapy, recorded 4 moderate AEs [61]. The reported injuries were related to overuse, such as joint/muscle pain and muscle stiffness. However, these side effects were transient, and patients fully recovered in a few days [61]. Considering the studies including patients with bone health impairments (e.g., osteoporosis or osteopenia), no AEs were registered [55,67,73]. Notably, no skeletal fractures have occurred neither in interventions involving high-impact training, such as that of *Taaffe* et al., which proposed for patients with prostate cancer (50% with osteopenia, 4% with osteoporosis) initiating ADT a six-month supervised aerobic nor during resistance training at high impact [73]. Considered comprehensively, exercise appears safe in this population; however, given the inconsistency in the collection and reporting of the AEs across the investigations, the abovementioned considerations made for the metastatic bone disease are relevant here as well.

### 4.2. Effect of Exercise on Bone Health

Exercise has been hypothesized as a strategy able to counteract the acceleration of bone loss due to cancer and its treatments. In this sense, a meta-analysis, including 26 randomized controlled trials, has demonstrated that exercise may produce significant improvements in bone-related outcomes, such as whole body, hip, trochanter, and femoral neck bone density among patients with cancer [25]. Analyzing the trials, which included patients at high risk of losing bone, some reported the inability of exercise to preserve bone in patients with cancer [56,65,66,68,70,71]. On the other hand, different investigations found improvements in bone mineral density among patients at high risk of losing bone tissue, even if considerable heterogeneity regarding the skeletal sites has been observed [59,60,62,64,69,74,75]. For instance, a 12-month randomized controlled trial, including 498 patients with breast cancer treated with chemotherapy and/or radiotherapy and/or undergoing endocrine treatments, has explored the impact of a supervised weekly aerobic or circuit training plus home-based, vigorous-intensity aerobic activity 2–3 times per week on bone tissue. Post-intervention evaluations revealed that compared to usual care, women in premenopausal status who performed the experimental intervention reported preservation in femoral neck bone mineral density (−0.2%, 95% CI: −0.9 to 0.6 vs. −1.4%, 95% CI: −2.1 to 0–07; *p* = 0.01), but not in the lumbar spine [59]. On the contrary, *Winters-Stone and colleagues* reported that 12 months of combined aerobic and resistance exercise intervention was able to improve lumbar spine body mass density (0.41 vs. −2.27; *p* = < 0.01), but not that of the femoral neck (−1.37 vs. −2.06; *p* = 0.27) in postmenopausal patients with breast cancer [60]. Focusing on studies that included patients with cancer and a diagnosed osteopenia or osteoporosis condition, only one investigation did not report improvements in terms of bone outcomes [67]. A 6-month exercise intervention, composed of a supervised and home-based aerobic training program performed 5 days per week, was shown to maintain bone mineral density in 75 postmenopausal women with breast cancer, 11% affected by osteopenia [55]. Another trial investigating 12 months of supervised and unsupervised strength training twice a week did not produce significant effects in terms of the bone mineral density of the spine and hip. However, the subtle changes in bone tissue were sufficient to produce a shift in the distribution of bone categories favoring the experimental group over the controls: a major number of women allocated in the usual care group became osteopenic at the spine compared to patients who performed the exercise program [63]. However, two main factors seem to influence the effectiveness of exercise in bone enhancement: adherence to exercise training and the timing of starting the exercise program with respect to endocrine therapy. For instance, a randomized controlled trial has investigated the effect of 24-month strength training on bone mineral density, in addition to calcium, vitamin D, and risedronate, in 249 patients with breast cancer affected by osteoporosis or osteopenia. The intention-to-treat analysis did not find significant differences in bone health improvement compared to controls that received medication alone. Per-protocol analysis revealed that those patients who attended at least ≥50% of the exercise sessions were less likely to lose bone than controls. In particular, in this subgroup of subjects, only 1.2% and 12.3% lost total hip and femoral neck bone mineral density, respectively, in contrast to controls, in which 8.6% and 26.7% reported a decrease in bone in the same skeletal sites [58]. Regarding the optimal timing for exercise initiation according to endocrine therapy, a study involving 104 patients with prostate cancer has explored if it is more efficacious to prevent bone loss using exercise from the start of ADT rather than trying to recover bone health initiating training after 6 months of endocrine therapy [73]. In this sense, a group was allocated to an immediate six-month supervised aerobic and resistance training, while the other was assigned to usual care followed by six months of the same training. Although total hip and whole-body bone mineral density declined similarly between the 2 groups, the spine bone mineral density was largely preserved in patients who engaged early in exercise (−0.4% vs. −1.6%), thus suggesting that exercising since the time of treatment may be more efficacious to prevent or attenuate the development of treatment-related side effects [73].

### 4.3. The Overall Effect of Exercise

Beyond the impact on bone health status, exercise may confer several other benefits to patients with cancer in this setting. Although not all the studies reported positive results on other outcomes, and most found no changes or even an increase in fat tissue [56,60,61,66], exercise may improve physical parameters, as well as patients’ psychological status and quality of life [62,65,66,71,72]. *Cormie and colleagues* proposed a supervised combined exercise program involving aerobic and strength sessions for 63 patients with prostate cancer scheduled to undergo ADT. After three months, compared to men allocated in the controls, those in the experimental arm experienced significant preservation in appendicular lean mass (mean difference 0.4, CI. 0.1 to 0.7, *p* = 0.01), a decrease in fat mass (mean difference −1.4, CI: −2.3 to −0.6, *p* = 0.001), and an increase in cardiorespiratory fitness (mean difference 1.1, CI: 0.4 to 1.9, *p* = 0.004) and strength. Additionally, the exercisers experienced improvements in treatment-related symptoms, fatigue, sexual activity and function, psychological status (distress and depression), and total cholesterol [65]. Similarly, another investigation on 100 patients with breast cancer in postmenopausal status, which tested 16 weeks of aerobic and strength training thrice a week, found similar results, e.g., improvements in cardiorespiratory fitness, muscle strength fatigue, depression, and quality of life [71]. However, most of the data come from studies that excluded patients affected by bone fragility conditions (osteopenia or osteoporosis), thus necessitating an expansion of research on the impact of exercise in these populations in the future.

## 5. Mechanisms by Which Exercise Improves Bone

Bone is a dynamic tissue that continuously undergoes remodeling throughout life, thanks to the constant activities of renewal and repair [76]. In this sense, bone homeostasis is strictly regulated by the well-balanced actions of osteoclasts, responsible for bone resorption, and osteoblasts involved in the formation of new bone. Whereas these two processes, if stable, guarantee a constant amount of bone, some conditions may impair the regulatory pathways shifting the balance towards an accelerated bone turnover (e.g., osteoporosis, bone metastases) and/or an increase bone production [76]. The main determinant of bone remodeling is represented by the mechanical stress (and, thus, the obtained tissue deformation—strain) induced by the loads carried by the bones. This system, known as “*mechanostat theory*”, involves bone cells that, if stimulated above a certain threshold of strain, react to strain, shifting the balance toward an increase in bone formation [77]. On the other hand, if the strain produced is lower than the homeostasis threshold, bone loss occurs [77].

In this context, exercise may produce an adequate load stimulus able to enhance bone formation, with deposition predominating over resorption [78]. However, not all the stimuli generated by exercise are similar and produce the same effects on bone turnover. For instance, activities with low/absent mechanical load, such as swimming and cycling, are unable to generate an adequate signal to shift the balance toward bone formation [79]. On the contrary, weight-bearing training, such as walking, stair climbing, and jogging, has been shown to have a great degree of load and, therefore, a greater capacity to induce osteogenesis. Bone modifications are site-specific and not systemic, in other words, a better anabolic response occurs in those skeletal sites subjected to a greater load [79]. Moreover, evidence states that bone mechanical loading is more effective if dynamic rather than static. In addition, the rate of applied strain affects the osteogenic capacity of exercise, i.e., bone responds better if loads are applied at a high rate [79]. In practice, exercises with high impact, e.g., those which include jumping, should be preferred to build bones, even if safety issues regarding these types of activities should always be kept in mind, especially in frail and elderly populations [79]. Finally, bone cells acquire desensitization to the mechanical loading immediately after a few repetitions; thus, inserting rest periods between exercises is the best way to maximize the anabolic response in bone [79].

**Figure 1:**
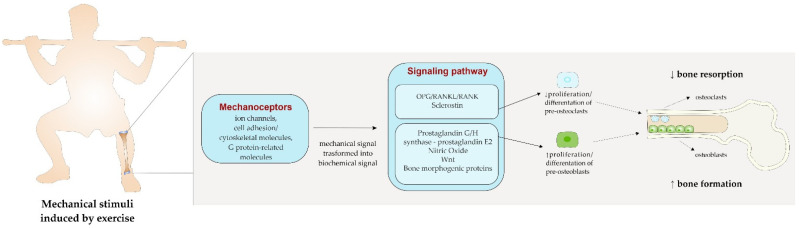
The effect of mechanical load induced by exercise on bone.

From a closer perspective (Figure 1), the load produced by exercise is usually perceived by ion channels, cell adhesion/cytoskeletal molecules, and G protein-related molecules, which are classified as mechanoreceptors in bone cells and translate the mechanical *stimuli* into biological signals [79]. Subsequently, a series of biochemical signaling have been identified as potential pathways to propagate the *stimuli* within cells and thus activate osteogenesis. In this sense, it has been found that mechanical stimulation activates the prostaglandin G/H synthase (or cyclooxygenase [COX])-prostaglandin E2(PGE2) and nitric oxide (NO) pathways, as well as the OPG/RANKL/RANK signaling pathways which, in turn, have been related to the suppression of bone resorption, and to enhancement in bone formation, thus favoring bone anabolic response [79]. PGE2 has been found to stimulate osteoblasts proliferation and differentiation. The mechanical load may increase osteocyte-derived PGE2 release and the expression of COX-2, the key enzyme involved in PGE2 production [78]. Conversely, NO exhibits dual effects on osteoblasts activity, depending on its concentration. A high dosage of NO induced by cytokine-stimulated cells inhibits bone formation by reducing osteoblasts’ proliferation, enhancing their apoptosis, and increasing the osteoclast-mediated resorption [80]. On the contrary, a low amount of NO, released by mechanically stimulated osteoblasts and osteocytes, has been shown to increase osteoblasts’ proliferation [80]. Exercise may enhance bone by regulating bone morphogenic proteins (BMP). BMP are members of the transforming growth factor beta (TGFβ) superfamily and are directly implied in osteoblastogenesis. The mechanical strain induced by exercise has been shown to upregulate several types of BMP, such as BPM-2, and BMP-7, which enhance the osteoblasts’ differentiation [81]. Moreover, the activity of osteoclasts is highly modulated by the OPG/RANKL/RANK signaling pathways. RANKL is a mediator produced by osteoblasts that can bind RANK, a specific receptor expressed on osteoclast progenitor cells and mature osteoclasts, which in turn enhances the transformation of mononuclear precursors into mature osteoclasts. The OPG, on the other hand, binds RANKL before its interaction with RANK, thus preventing osteoclast differentiation. Exercise acts on this pathway by increasing the level of OPG and reducing the expression of RANKL, finally resulting in an inhibition of osteoclasts’ differentiation and activity [82]. Another pathway triggered by exercise load and suggested as the major contributor to bone cell mechanotransduction is the Wnt signaling pathway [83]. The Wnt pathway modulates the expression of osteoblastic factors which, through the stimulation of the mesenchymal stem cells, promotes the proliferation and differentiation of osteoblast precursors. Moreover, Wnt signaling is also implied in the downregulation of osteoclastic activity and osteoclastogenesis, slowing down bone resorption [83]. The activity of the Wnt signaling is highly modulated by sclerostin, a protein produced by the SOST gene, which inhibits the pathway, thus reducing osteoblastogenesis and bone formation [83]. Mechanical loading can downregulate the sclerostin expression in bone, allowing for the subsequent activation of the Wnt pathway, thereby increasing bone formation and decreasing the resorption through the inhibition of osteoclast activity [84].

In a more indirect manner, exercise may favor bone anabolic response through the modulation of the inflammatory status. Indeed, emerging evidence suggests that inflammation may elicit a direct impact on bone turnover. The effect of inflammatory processes on bone has been described in several chronic inflammatory diseases, such as periodontitis, rheumatoid arthritis, aseptic prosthesis loosening, and chronic obstructive pulmonary disease [85]. Tumor-promoting inflammation is a hallmark of cancer, making cancer a full-fledged inflammatory disease [86]. Although the exact mechanisms by which inflammation may regulate bone remodeling remain to be elucidated, several cytokines and growth factors have been shown to regulate the osteoblasts’ and osteoclasts’ activity [87]. Some inflammatory mediators, including IL-1, IL-6, and IL-11, may act through the OPG/RANKL/RANK pathway, upregulating the RANKL expression and thus stimulating osteoclastogenesis [88], while others, such as TNF-α, may impair bone remodeling through the disruption of osteoblasts’ differentiation [85]. On the other hand, other cytokines, such as IL-4 and IFN-gamma, have demonstrated an inhibitory effect on osteoclasts’ differentiation, even if it is often overshadowed by those which promote osteoclasts’ activation [85].

## 6. Consideration about Exercise Prescription in Patients with Bone Metastases or with Osteoporosis

Exercise is a crucial intervention in the oncological setting, able to improve physical parameters and counteract treatment-related side effects [18]. In patients with cancer affected by bone impairments, exercise is able to increase physical function, enhance quality of life and potentially improve bone health. However, to prescribe and deliver a safe and feasible exercise program in a frail population and prevent/reduce the risk of SREs and fractures related to osteoporosis, some considerations should be applied. 

To date, specific screening tools to determine the risk and benefit *ratio* of an exercise program in this setting are currently absent [89]. In this situation, thus, patient assessment is a crucial step for obtaining relevant information to program a safe and personalized exercise program. Beyond the patient’s medical history and anticancer treatment plan, physical and psychological evaluation, including cardiorespiratory fitness, strength, body composition, as well as the barriers and preferences experienced during exercising, allow consideration of the expected heterogeneity among patients [90]. In accordance with the current exercise recommendations for people with bone metastases, physical testing should be adapted (e.g., avoiding tests that apply a high load on metastatic bone sites) [89]. In this sense, the assessment of bone health status is fundamental. Acquiring information regarding the severity of bone impairments, e.g., the status of osteopenia or osteoporosis in non-metastatic patients, as well as the number, type, size, and location of skeletal lesions in people with bone metastases, is essential to target exercise testing and programming [89]. Additionally, whereas osteoporosis is often painless, pain at rest or during movements at the skeletal lesion is one of the most common symptoms in patients with bone metastases [91]. Evaluation of pain, e.g., using the brief pain inventory or visual analogue scale, may be useful to establish its severity [91]. Since pain during functional activity is associated with increased fracture risk, it should also be strictly monitored during exercise [91].

In 2019, the American College of Sports Medicine updated the guidelines for exercise in people with cancer [18]. These recommendations suggest that patients with cancer should engage in moderate-intensity aerobic activity, at least 30 min per session, and resistance training, i.e., contracting the muscles against a resistance to overload and bring about a training effect in the muscular system, 2 times per week, utilizing 2 sets of 8–15 repetitions at moderate intensity [18]. In addition to these cancer-specific exercise guidelines, recommendations about exercise in osteoporotic people may offer additional guidance. Particularly, balance training, i.e., exercises aiming to improve controls of rapid balance reaction, can be included in the exercise sessions in order to prevent falls and, thus, the fracture risk [52,92]. Clearly, these recommendations are general, and exercise should be personalized, taking into account the effect on bone remodeling on the one hand, and safety issues on the other. For instance, concerning aerobic activity, training on a treadmill produces a greater bone anabolic effect compared to cycling; nevertheless, walking on a treadmill may expose patients to higher risk of falls than cycling. A similar comparison could be applied to resistance training. Free-weight resistance training or high-impact training (e.g., jumping) may be more effective in increasing mechanical load and, thus, bone formation with respect to other forms of strength training, e.g., those with isotonic machines or with elastic bands. Even in this case, free-weight resistance and high-impact training may have a greater risk of injury than other activities. To cope with these complex situations, as advised by the International Bone Metastases Exercise Working Group, the prescription and delivery of exercise should be performed by university-qualified exercise professionals who have additional cancer exercise education and appropriate experience in working with patients with bone metastases [89]. These experts possess the professional expertise to adequately weigh the risk–benefit ratio of testing and exercise programs/activities based on the patient’s condition, and may offer appropriate monitoring of the patient’s exercise response while paying attention to the correct exercise technique on postural alignment [89]. A final consideration to keep in mind is related to the fact that most patients might be highly deconditioned. In this sense, it might be necessary to start with a low dose of exercise and progressively increase it over the weeks, according to the patient’s response.

## 7. Conclusions

According to the available evidence, exercise may offer a safe approach to improving physical function and self-reported outcomes, and to potentially enhance bone health in patients with cancer affected by bone impairments. Although some trials are currently ongoing to enrich the currently available and evidence-based data (Table 3.), additional studies are needed in order to consolidate the impact of exercise on safety and bone outcomes, as well as to develop adequate tools to screen patients’ eligibility for exercise intervention. Based on the currently available evidence, exercise has been shown to be safe and feasible in patients with cancer suffering from bone metastases, affected by osteoporosis/osteopenia, or at risk of bone loss. Moreover, exercise may help improve bone health, physical function, and quality of life, and help manage cancer and treatment-related side effects. Therefore, patients should be supported to engage in sufficient physical activity and encouraged to include in their exercise routine those activities that may favor a bone anabolic response. Nevertheless, given the recognized peculiarity of this population, the prescription and delivery of exercise should be performed by suitable experts who have specific training in these settings.

## Figures and Tables

**Table 1:** Overview of the randomized controlled studies conducted on patients with bone metastases.

Author (Year)	Sample Size (% BM), Cancer Type	Exercise Intervention	Recruitment and Adherence Rate	Dropout Rate	Safety	Main Findings
Cormie et al. [26] (2013) RCT	20 pts (100%) with metastatic prostate cancer and mixed treatment status	12 weeks of supervised resistance training at moderate intensity twice a week (EX) vs. usual care (CG)	RR: 74% AR: 83%	Exp: 0% Ctrl: 0%	Exp: 0 Ctrl: 0	↑ EX vs. CG in strength, lean body mass, aerobic capacity, amount of physical activity ↔ EX vs. CG in fat mass, balance, fatigue, QoL, psychological distress
Litterini et al. [27] (2013) RCT	66 pts (24.2%) with mixed cancers and mixed treatment status	10 weeks of supervised resistance training at moderate intensity (RT) vs. supervised aerobic training (AT) at moderate/vigorous intensity	RR: NR AR: NR	RT: 32.6% AT: 9.4%	Exp: 0 Ctrl: 0	↑ functional mobility, gait speed, fatigue in RT group ↑ functional mobility, gait speed, fatigue in AT group
Rief et al. [28,29,30,31,32] (2014–2016) RCT	60 pts (100%), with mixed cancers, undergoing radiotherapy	2 weeks of supervised resistance training 5 days per week + home-based resistance training 3 days per week until 6 months (EX) vs. passive physical therapy (CG)	RR: 75% AR: NR	Exp: 50% (death) Ctrl: 40% (death)	Exp: 0 Ctrl: 0	↑ EX vs. CG in the assumption of oral morphine equivalent dose, pain medication, pain level, bone density of metastatic sites, bone density of osteolytic metastases, psychosocial aspects, physical fatigue, interference with daily life, emotional distress, bone local progression ↔ EX vs. CG neuropathic pain, bone density of osteoblastic metastases, overall and bone survival, progression-free survival, pathological fractures, emotional and cognitive fatigue, social sequelae
Uth et al. [33,34] (2014–2016) RCT	57 pts (19.3%) with advanced prostate cancer and mixed treatment status	12 weeks of football 2–3 times per week (EX) vs. usual care (CG)	RR: 15% AR: 76.5%	Exp: 10.3% Ctrl: 17.8%	Exp: 2 fibula fractures; 1 partial rupture of the Achilles tendon; 1 ankle strain; 1 quadriceps muscle strain related to exercise Ctrl: NR	↑ EX vs. CG in bone mineral content (total and leg), procollagen type 1 amino-terminal propeptide, lean body mass, strength, ↔ EX vs. CG in bone mineral density, balance, fat mass, waist-to-hip ratio, cardiorespiratory fitness
Uth et al. [35] (2016) RCT	57 pts (19.3%) with advanced prostate cancer and mixed treatment status	32 weeks of football 2–3 times per week (EX) vs. usual care (CG)	RR: 15% AR: 46.2%	Exp: 27.6% Ctrl: 28.6%	Exp: 2 fibula fractures; 1 partial rupture of the Achilles tendon; 1 ankle strain; 1 quadriceps muscle strain related to exercise Ctrl: NR	↑ EX vs. CG in hip and femoral shaft bone mineral density, osteocalcin level, stair-climbing performance ↔ EX vs. CG in bone mineral density of femoral neck and spine, lean body mass, fat mass, strength
Galvao et al. [36] (2017) RCT	57 pts (100%) with metastatic prostate cancer and mixed treatment status	3 months of supervised aerobic and resistance training thrice a week (EX) vs. usual care (CG)	RR: 55.3% AR: 89%	Exp: 17.9% Ctrl: 10.3%	Exp: 0 Ctrl: 0	↑ EX vs. CG in physical function, strength ↔ EX vs. CG in balance, lean mass, fat mass, fatigue, bone pain, cardiorespiratory fitness
Rosenberg et al. [37] (2017) Two-arm non RCT	20 pts (30%), with mixed cancers undergoing tyrosine kinase inhibitors	12 weeks of supervised resistance training, twice a week (EX) vs. usual care (CG)	RR: 81.4% AR: 81%	Exp: 9% Ctrl: 28.6%	Exp: 11 non-serious AEs weakness, join pain, hernia inguinalis not related to exercise; 1 pts non-serious AEs nausea, vomiting, join pain during exercise Ctrl: NR	↑ EX vs. CG in strength (isometric knee extensors) ↓ EX vs. CG in dyspnea ↔ EX vs. CG in fatigue, motivation, depression, QoL, strength (isokinetic knee extensors, elbow flexors) and cardiorespiratory fitness
Solheim et al. [38] (2017) RCT	46 pts (17.4%) with lung or pancreatic cancer undergoing chemotherapy	6 weeks of aerobic training twice a week and resistance training thrice a week (EX) vs. usual care (CG)	RR: 11.5% AR: 60%	Exp: 8% Ctrl: 14.3%	Exp: 13 serious AEs, pain, neutropenia, infection, rectal bleeding not related to exercise Ctrl: 8 serious AEs, pain, neutropenia, GI stricture	↑ EX vs. CG in body weight ↔ EX vs. CG in muscle mass, amount of physical activity, 6MWT, strength, fatigue
Dawson et al. [39] (2018) RTC	37 pts (35%) with prostate cancer and mixed treatment status	12 weeks of supervised resistance training at moderate/vigorous intensity thrice a week (EX) vs. *stretching* (CG)	RR: 40.7% AR: 93.8%	Exp: 18.8% Ctrl: 9.5%	Exp: 0 Ctrl: 0	↑ EX vs. CG in sarcopenia prevalence, muscle mass, lean body mass, fat-free mass, waist circumference, strength, prostate cancer symptoms, QoL ↔ EX vs. CG in the prevalence of metabolic syndrome, insulin level, HOMA-IR, cholesterol, triglycerides, glucose, cardiorespiratory fitness, fatigue, depression, balance
Bjerre et al. [40] (2019) RCT	214 pts (19%) with prostate cancer and mixed treatment status	6 months of football twice a week (EX) vs. usual care (CG)	RR: 46.6% AR: NR	Exp: 4.8% Ctrl: 8.3%	Exp: 10 falls, 1 bone fracture and 11 hospital admissions not related to exercise; 58 muscle strains or sprains and 2 ruptures of the Achilles tendon related to exercise Ctrl: 6 falls, 2 bone fractures and 22 hospital admissions.	↑ EX vs. CG in mental health ↔ EX vs. CG in QoL, amount of physical activity, lean body mass, fat mass, bone mineral density, bone mineral content, physical health
Bjerre et al. [41] (2019) RCT	214 pts (19%) with prostate cancer and mixed treatment status	6 months of football twice a week (EX) vs. usual care (CG)	RR: 46.6% AR: NR	Exp: 4.8% Ctrl: 8.3%	Exp: 13 falls, 2 bone fractures Ctrl: 10 falls, 2 bone fractures	↑ EX vs. CG in hip bone mineral density at 1-year follow-up ↓ fat mass and hospital admission at 1-year follow-up ↔ EX vs. CG in spine bone mineral density, mental health, fat mass, lean body mass, QoL at 1-year follow-up
Cheville et al. [42] (2019) RCT	516 pts (51.3%) with mixed cancer and mixed treatment status	6 months of telerehabilitation composed of walking and resistance training (ARM1) vs. telerehabilitation + pharmacological pain management (ARM2) vs. usual care (CG)	RR: 6.7% AR: NR	Exp (ARM1): 13.4% Exp (ARM2): 16.9% Ctrl: 12.8%	Exp: NR Ctrl: NR	↑ AMR1 vs. CG in physical function, pain interference/intensity, QoL, number and length of hospital admissions ↑ AMR2 vs. CG in pain interference/intensity, number and length of hospital admissions ↔ AMR2 vs. CG in physical function
Sprave et al. [43] (2019) RCT	60 pts (100%) with mixed cancers undergoing radiotherapy	Supervised resistance training 5 days per week + home-based resistance training 3 days per week until 6 months (EX) vs. muscle relaxation (CG)	RR: 53.1% AR: 67% pts completed ≥80% of the supervised exercise; 64% pts completed ≥80% of the home-based exercise	Exp: 73% (most for death) Ctrl: 63% (most for death)	Exp: 0 Ctrl: 0	↔ EX vs. CG in pain, opioid consumption, bone density of metastases, QoL, distress, fatigue, pathological fracture
Villumsen et al. [44] (2019) RCT	46 pts (34.8%) with advanced prostate cancer and mixed treatment status	12 weeks of unsupervised, home-based aerobic and strength activity exergaming (Xbox 360) thrice a week (EX) vs. usual care (CG)	RR: 37.1% AR: NR	Exp: 8.7% Ctrl: 13%	Exp: 1 severe non-heart chest pain due to surgical clips in the thorax related to exercise Ctrl: 0	↑ EX vs. CG in 6MWT ↔ EX vs. CG in QoL, muscle power, fatigue, lean body mass, fat mass, amount of physical activity
Yee et al. [45] (2019) RCT	14 pts (35.7%) with metastatic breast cancer undergoing treatments (78.6%)	8 weeks of supervised resistance training at moderate intensity twice a week and walking activity at moderate intensity 5 days per week (EX) vs. no advice on exercise (CG)	RR: 93% AR: 100% for supervised sessions; 25% for walking	Exp: 0% Ctrl: 17%	Exp: 0 Ctrl: 0	↑ EX vs. CG in fatigue, pain, QoL (physical, role, emotional and social), 6MWT, cardiorespiratory fitness, ↔ EX vs. CG in strength, weight, and amount of physical activity
Bjerre et al. [46] (2021) RCT	41 pts (100%) with prostate cancer and mixed treatment status	6 months of football twice a week (EX) vs. usual care (CG)	RR: NR AR: 54%	Exp: 9% Ctrl: 16%	Exp: 1 fall Ctrl: 1 fall, 7 hospital admissions	↑ EX vs. CG in QoL, disease progression ↔ EX vs. CG in lean body mass, fat mass, bone mineral density, physical and mental health
Dalla Via et al. [47] (2021) RCT	70 pts (29%) with prostate cancer undergoing treatments	12 months of gym-based resistance and weight-bearing training twice a week + home-based resistance and weight-bearing training once a week (EX) vs. usual care (CG)	RR: 30.7% AR: 56%	Exp: 8.8% Ctrl: 19.4%	Exp: 21 non-serious musculoskeletal complaints related to exercise Ctrl: NR	↑ EX vs. CG in lower limb strength, dynamic mobility ↔ EX vs. CG in bone mineral density (all skeletal sites), bone strength, bone structure, body composition, upper limb strength
Galvao et al. [48] (2022) RCT	57 pts (100%) with metastatic prostate cancer and mixed treatment status	3 of months of supervised aerobic and resistance training thrice a week (EX) vs. usual care (CG)	RR: 55.3% AR: 89%	Exp: 17.9% Ctrl: 10.3%	Exp: 0 Ctrl: 0	↔ EX vs. CG in sexual function and capacity and in urinary and bowel function

Abbreviations: ↑, significant improvement; ↓, significant worsening; ↔, no significant change; EX, exercise group; CG, control group; BM, bone metastasis; RCT, randomized controlled trial; EX, exercise; NR, not reported; RR, recruitment rate; AR, adherence rate; Exp, experimental; Ctrl, control; 6MWT, six minutes walking test; QoL, quality of life; AEs, adverse events; GI, gastro-intestinal; HOMA-IR, homeostatic model assessment for insulin resistance.

**Table 2:** Overview of the randomized controlled studies conducted on patients with cancer affected by osteopenia/osteoporosis or at risk of bone loss.

Author (Year)	Sample Size and Cancer Type	Exercise Intervention	Recruitment and Adherence Rate	Dropout Rate	Safety	Main Findings
Schwartz et al. [54] (2007) RCT	66 pts with breast cancer undergoing doxorubicin or methotrexate at risk of bone loss	6 months of home-based aerobic training at moderate intensity 4 days a week (AT) vs. home-based resistance exercise (RT) vs. usual care (CG)	RR: 86.8% AR: NR	Exp: 0% Ctrl: 0%	Exp: NR Ctrl: NR	↑ AT vs. RT and CG in lumbar spine bone mineral density, aerobic capacity, strength
Irwin et al. [55] (2009) RCT	75 pts with breast cancer in postmenopausal status who completed chemo or radiotherapy and affected by osteopenia (11%)	6 months of supervised and home-based aerobic and resistance training at aerobic intensity twice a week (EX) vs. usual care (CG)	RR: 9.5% AR: NR	Exp: 32.4% Ctrl: 39.5%	Exp: NR Ctrl: NR	↑ EX vs. CG in body fat, lean body mass, total physical activity, daily steps ↔ EX vs. CG in weight, bone mineral density, bone mineral content, waist and hip circumferences ↑ EX vs. CG body fat, bone mineral density at 1-year follow-up ↔ EX vs. CG in weight, lean body mass, bone mineral content at 1-year follow-up Hormone therapy modified the effect of exercise on fat mass; age modified the effect of exercise on lean body mass and bone mineral density; disease stage modified the effect of exercise on fat mass, bone mineral density, and bone mineral content; adherence to exercise modified the effect of exercise on fat mass and bone mineral density;
Rogers et al. [56] (2009) RCT	41 pts with breast cancer taking AI or selective estrogen receptor modulators at risk of bone loss	12 weeks of physical activity behavior change intervention, including supervised and home-based training at moderate intensity (EX) vs. usual care (CG)	RR: 34% AR: 99%	Exp: 5% Ctrl: 5%	Exp: 0 Ctrl: 0	↑ EX vs. CG in the amount of physical activity, strength, social well-being, and joint stiffness, waist-to-hip ratio ↔ EX vs. CG in BMI, fat mass, femoral neck bone mineral density, and lumbar spine bone mineral density, perceived health, fatigue, endocrine symptoms, sleep domains
Twiss et al. [57] (2009) RCT	223 pts with breast cancer affected by OP (29%) or osteopenia (71%)	9 months of home-based resistance training + 15 months of supervised resistance training (EX) vs. usual care (CG)	RR: NR AR: 24–31%	Exp: NR Ctrl: NR	Exp: NR Ctrl: NR	↑ EX vs. CG in strength, balance ↔ EX vs. CG in falls, fractures
Waltman et al. [58] (2010) RCT	249 pts with breast cancer affected by OP or osteoporosis	24 months of supervised resistance training twice a week + calcium, vitamin D, and risedronate (EX) vs. calcium, vitamin D, and risedronate (CG)	RR: 35.2% AR: 69.4%	Exp: 10.5% Ctrl: 9.6%	Exp: NR Ctrl: NR	↑ EX vs. CG in strength ↔ EX vs. CG in bone mineral density, calcium level, Alkphase B, Serum NTX Per protocol analysis showed that subjects with 50% greater adherence to exercise intervention were less likely to lose bone mass density at the total hip and femoral neck
Saarto et al. [59] (2011) RCT	498 pts with breast cancer, pre- or postmenopausal status undergoing endocrine therapy at risk of bone loss	12 months of supervised aerobic or circuit training one a week and home-based aerobic training at vigorous intensity 2–3 times a week (EX) vs. usual care (CG)	RR: 78% AR: 58% premenopausal pts; 63% postmenopausal pts	Exp: 7.3% Ctrl: 6.3%	Exp: NR Ctrl: NR	↑ EX vs. CG in femoral neck bone mineral density in the premenopausal group ↔ EX vs. CG in lumbar spine bone mineral density, femoral neck bone mineral density in the postmenopausal group, bone mineral content, lean body mass, fat mass, amount of physical activity
Winters-Stone et al. [60] (2011) RCT	106 pts with breast cancer in postmenopausal status at risk of bone loss	12 months of moderate intensity resistance and impact training, twice a week supervised + once a week home-based session (EX) vs. flexibility training (CG)	RR: 29.5% AR: 57% for exercise group; 62% for flexibility	Exp: 30.8% Ctrl: 42.6%	Exp: 0 Ctrl: 0	↑ EX vs. CG in lumbar spine bone mineral density, osteocalcin ↔ EX vs. CG in fat mass, lean body mass, hip bone mineral density, trochanter bone mineral density, femoral neck bone mineral density, deoxypyridinoline
Nikander et al. [61] (2012) RCT	86 pts with breast cancer undergoing endocrine therapy at risk of bone loss	12 months of home-based impact aerobic training thrice a week + a weekly supervised group training at vigorous intensity (EX) vs. usual care (CG)	RR: NR AR: 76%	Exp: 18.9% Ctrl: 7.5%	Exp: 4 moderate overuse injuries (joint and muscle pain, muscle stiffness) Ctrl: 0	↑ EX vs. CG in bone structural strength, strength, agility ↔ EX vs. CG in bone mineral content, fat mass, jump force, 2 km walking time
Winters-Stone et al. [62] (2013) RCT	71 pts with breast cancer in postmenopausal status at risk of bone loss	12 months of moderate intensity resistance and impact training, twice a week supervised + once a week home-based session (EX) vs. flexibility training (CG)	RR: 27.5% AR: 84% for exercise group; 100% for flexibility	Exp: 34.3% Ctrl: 30.6%	Exp: NR Ctrl: NR	↑ EX vs. CG in upper strength, femoral neck and spine bone mineral density in women who were 1+ year past the onset of menopause ↔ EX vs. CG in bone mineral density (all skeletal sites), osteocalcin, lean mass, deoxypyridinoline, fat mass
Winters-Stone et al. [63] (2014) RCT	258 pts with breast cancer affected by osteopenia or OP	3 months of supervised strength training twice a week + 9 months of unsupervised strength training (EX) vs. usual care (CG)	RR: 9.2% AR: 72%	Exp: 14% Ctrl: 12%	Exp: NR Ctrl: NR	↔ EX vs. CG in bone mineral density (all skeletal sites) A significant number of postmenopausal women in the controls became osteogenic compared to those in the exercise group
Winters-Stone et al. [64] (2014) RCT	51 pts with prostate cancer taking ADT at risk of bone loss	12 months of moderate intensity resistance and impact training, twice a week supervised + once a week home-based session (EX) vs. flexibility training (CG)	RR: 10.9% AR: 84% supervised/43% home-based for resistance training; 74% supervised/51% home-based for flexibility	Exp: 10% Ctrl: 16%	Exp: 0 Ctrl: 0	↑ EX vs. CG in bone mineral density of lumbar vertebrae 4 ↔ EX vs. CG in bone mineral density of lumbar vertebra 1, 2, and 3, in osteocalcin, deoxypyridinoline
Cormie et al. [65] (2015) RCT	63 pts with prostate cancer and initiating ADT at risk of bone loss	3 months of supervised aerobic and resistance training at moderate-high intensity twice a week (EX) vs. usual care (CG)	RR: 50% AR: 95.8%	Exp: 3.1% Ctrl: 22.6%	Exp: 0 Ctrl: NR	↑ EX vs. CG in total and appendicular lean mass, physical/mental functioning, cardiorespiratory fitness, fat mass, trunk fat mass, HDL-cholesterol, sexual function, fatigue, psychological distress ↔ EX vs. CG in hip bone mineral density, lumbar spine bone mineral density, whole-body bone mineral density or tibia bone mineral density, markers of chronic disease risk and bone formation/resorption
Nilsen et al. [66] (2015) RCT	58 pts with prostate cancer undergoing ADT at risk of bone loss	16 weeks of high-load strength training thrice a week (EX) vs. usual care (CG)	RR: 48.7% AR: 88% lower body exercises; 84% upper body exercises	Exp: 21.4% Ctrl: 10.0%	Exp: NR Ctrl: NR	↑ EX vs. CG lean body mass of the lower and upper extremities, appendicular lean body mass, strength, cardiorespiratory fitness ↔ EX vs. CG in total and trunk lean body mass, trunk fat mass, fat percentage, body mass, bone mineral density (all skeletal sites), QoL
Kim et al. [67] (2016) RCT	43 pts with breast cancer affected by osteopenia (100%)	6 months of home-based aerobic and resistance training + calcium and vitamin D (EX) vs. calcium and vitamin D (CG)	RR: 19.5% AR: 69.5% resistance training; 48.5% aerobic training	Exp: 13% Ctrl: 5%	Exp: 0 Ctrl: 0	↔ EX vs. CG in bone mineral density (all skeletal sites), calcium level, type I collagen linked N-telopeptide, 6MWT, amount of physical activity, strength
Knobf et al. [68] (2016) RCT	154 pts (women) with mixed cancer types affected in postmenopausal status at risk of bone loss	12 months of supervised aerobic and resistance training at moderate intensity thrice a week + calcium and vitamin D (EX) vs. home-based physical activity + calcium and vitamin D (CG)	RR: 22.9% AR: 77.4%	Supervised: 18% Home-based: 18%	Supervised: NR Home-based: NR	↑ EX vs. CG in the amount of physical activity ↔ EX vs. CG in bone mass density (all skeletal sites), osteocalcin Bone loss was higher in pts using AI compared to pts taking tamoxifen or no endocrine therapy independently from exercise intervention
Kim et al. [69] (2017) RCT	51 pts with prostate cancer undergoing ADT at risk of bone loss	6 months of home-based program including weight bearing, calisthenics, resistance, and balance exercises at moderate intensity + DVD material + educational sessions (EX) vs. stretching (CG)	RR: 14.0% AR: 84.7% weight bearing exercises; 64.8% resistance exercises; 40% stretching group	Exp: 11.9% Ctrl: 28.0%	Exp: 0 Ctrl: 0	↑ EX vs. CG in left grip strength, ↔ EX vs. CG in bone mass density (all skeletal sites), QoL, serum NTX, BS-ALP
De Paulo et al. [70] (2018) RCT	36 pts with breast cancer in postmenopausal status undergoing AI at risk of bone loss	36 weeks of aerobic and resistance training at moderate intensity thrice a week + education lectures once a month (EX) vs. stretching and relaxation exercises (CG)	RR: 10.3% AR: 83%	Exp: 16.7% Ctrl: 22.2%	Exp: NR Ctrl: NR	↑ EX vs. CG in fat mass, trunk fat mass ↔ EX vs. CG in bone mineral density (all skeletal sites), lean mass, cholesterol, triglycerides glucose, C-reactive protein, CTX
Dieli-Conwright et al. [71] (2018) RCT	100 pts with breast cancer in postmenopausal status (60%) at risk of bone loss	16 weeks of supervised aerobic and resistance training thrice a week (EX) vs. usual care (CG)	RR: 23.9% AR: 95%	Exp: 8% Ctrl: 10%	Exp: 0 Ctrl: 0	↑ EX vs. CG in cardiorespiratory fitness, strength, QoL, fatigue, depression Significant increase EX vs. CG in osteocalcin, alkaline phosphatase ↔ EX vs. CG in bone mineral density (all skeletal sites), CTX, NTX, RANK, RANKL
Thomas et al. [72] (2018) RCT	121 pts with breast cancer in postmenopausal status undergoing AI at risk of bone loss	12 months of supervised resistance training twice a week + 150 min of moderate-intensity aerobic training at home (EX) vs. usual care (CG)	RR: 11.9% AR: NR	Exp: 21.3% Ctrl: 35%	Exp: NR Ctrl: NR	↑ EX vs. CG in cardiorespiratory fitness, the amount of physical activity, lean body mass, join pain ↔ EX vs. CG in body fat, bone mineral density
Taaffe et al. [73] (2019) RCT	104 pts with prostate cancer affected by osteopenia (50%) or OP (4%) and initiating ADT	6 months of supervised aerobic, resistance, and impact loading training thrice weekly + 6 months of usual care + daily calcium and vitamin D (IMEX) vs. 6 months of usual care + 6 months of supervised aerobic, resistance, and impact loading training thrice weekly + daily calcium and vitamin D (DEL)	RR: 47.5% AR: 79% for IMEX; 69% for DEL	IMEX: 13% DEL: 36%	IMEX: 0 DEL: 0	Preservation in lumbar spine bone mineral density during the exercise period in IMEX and DEL ↑ IMEX vs. DEL in lean mass, appendicular skeletal muscle, and muscle density at 6 months DEL recovered lean mass, appendicular skeletal muscle, and muscle density at 12 months ↔ IMEX vs. DEL in fat mass, trunk fat mass PSA, testosterone, P1NP, ALP, NTX, at 6 and 12 months
Tabatabai et al. [74] (2019) RCT	206 pts with breast cancer in premenopausal status who received adjuvant chemotherapy at risk of bone loss	12 months of partially supervised aerobic and resistance training thrice a week at moderate intensity (EX) vs. usual care (monthly newsletter) (CG)	RR: NR AR: NR	Exp: 8.7% Ctrl: 8.7%	Exp: 1 non-serious (nasal discharge) Ctrl: NR	Significant increase in lumbar spine bone mineral density in pts performed exercise and who preserved lean mass ↔ EX vs. CG in lumbar spine bone mineral density, osteocalcin, P1NP, C-telopeptides, N-telopeptides, free testosterone, estrone, estradiol, 25-hydroxyvitamin D, HOMA-IR, serum fructosamine, cholesterol
Uth et al. [75] (2021) RCT	68 pts with breast cancer in pre- or postmenopausal status, undergoing AI, tamoxifen or trastuzumab at risk of bone loss	12 months of football 2–3 times per week (EX) vs. usual care (CG)	RR: 83.9% AR: 44%	Exp: 28.0% Ctrl: 27.0%	Exp: 15 non-serious musculoskeletal trauma or overload related to exercise Ctrl: 2 musculoskeletal trauma	↑ EX vs. CG in lumbar vertebrae 1 and 4 bone mineral density, strength, postural balance ↔ EX vs. CG in total bone mineral density, total hip bone mineral density, femoral neck bone mineral density, trochanter bone mineral density, femoral shaft bone mineral density, BMI, lean body mass, fat mass, CTX, osteocalcin, P1PN

Abbreviations: ↑, significant improvement; ↓, significant worsening; ↔, no significant change; OP, osteoporosis; RCT, randomized controlled trial; EX, exercise; NR, not reported; RR, recruitment rate; AR, adherence rate; Exp, experimental; Ctrl, control; BMD, bone mineral density; BMI, body mass index; ADT, androgen-deprivation therapy; AI, aromatase inhibitors; PSA, prostatic specific antigen; P1NP, procollagen type 1 N-terminal propeptide; ALP, alkaline phosphatase; NTX, N-terminal telopeptide of type 1 collagen; CTX, C-telopeptide of type 1 collagen, RANK, receptor activator factor-kappa B; RANKL, receptor activator factor-kappa B ligand; HOMA-IR, homeostatic model assessment for insulin resistance.

**Table 3:** Randomized controlled trials currently ongoing in patients with bone metastases or in patients with cancer affected by osteoporosis/osteopenia or at risk of bone loss.

PI and Sponsor	Number	Title	Intervention	Study Design and Population	Primary Outcome	Secondary Outcomes
*Ongoing studies on patients with cancer affected by osteoporosis/osteopenia or at risk of bone loss*						
Michael Harrison, University of Pittsburgh	NCT05156424	A Comparison of Aerobic and Resistance Exercise to Counteract Treatment Side Effects in Men with Prostate Cancer	6 months of aerobic exercise intervention vs. resistance intervention	RCT on 24 pts with prostate cancer undergoing androgen deprivation therapy	Feasibility	QoL, fatigue, amount of physical activity, anthropometric measure, body composition, bone mineral density, arterial stiffness, cardiovascular fitness, strength, balance, 6MWT, flexibility, blood biomarkers
Luke J. Peppone, University of Rochester	NCT01419730	Vitamin D and Physical Activity on Bone Health	6 months of vitamin D + walking and resistance training vs. vitamin D vs. usual care	RCT on 191 pts with breast cancer undergoing aromatase inhibitors	Bone mineral density	Balance, aerobic capacity, and strength
Catherine L. Carpenter, Jonsson Comprehensive Cancer Center	NCT03953157	Dietary and Exercise Interventions in Reducing Side Effects in Patients with Stage I-IIIa Breast Cancer Receiving Aromatase Inhibitors	3 months of dietary intervention vs. exercise intervention	RCT on 20 pts with breast cancer undergoing aromatase inhibitors	Bone mineral density	Joint and muscle pain, inflammatory markers
*Ongoing studies on patients with bone metastases*						
Manuel Arroyo-Morales, University of Granada	NCT05244382	Overcome, a Program of Therapeutic Exercise and Functional Recovery to Improve the Functional Capacity of Women with Breast Cancer and Bone Metastases	3 months of aerobic exercises on antigravity treadmill + functional recovery of motor control with feedback ultrasound and occupational therapy vs. usual care	RCT on 58 patients with breast cancer and bone metastases	6MWT, QoL	Strength, body composition, muscle architecture, pain, anxiety and depression, opioid consumption

Abbreviation: 6MWT, six minutes walking test; QoL, quality of life; RCT, randomized controlled trial.
